# Accidental Intravenous Administration of Simethicone in a 4-Year-Old Patient: A Case Report

**DOI:** 10.15388/Amed.2024.31.1.19

**Published:** 2024-02-27

**Authors:** Agnė Lozovskytė, Robertas Badaras, Gabija Laubner Sakalauskienė

**Affiliations:** 1 Faculty of Medicine, Vilnius University, Vilnius, Lithuania; 2 Clinic of Anaesthesiology and Intensive Care, Centre of Toxicology Faculty of Medicine, Vilnius University, Vilnius, Lithuania; 3 Faculty of Medicine, Vilnius University, Vilnius, Lithuania Toxicology Centre, Republican Vilnius University Hospital, Vilnius, Lithuania

**Keywords:** Simethicone, Accidental intravenous administration, Respiratory distress ir pediatric case, Medication error, Simetikonas, Netyčinis intraveninis skyrimas, Pediatrinis respiracinio distreso sindromo atvejis, Vaistų skyrimo klaida

## Abstract

Simethicone is an antiflatulent medication exclusively administered orally, thus its systemic effects remain unknown. We present a case of an inadvertent intravenous administration of simethicone to a 4-year-old patient, precipitating respiratory difficulty, cyanosis, and altered mental status. The patient’s condition improved rapidly with appropriate interventions, leading to discharge in a fully recovered state. To date, only one documented instance of intravenous simethicone administration exists in medical literature.

## Abstract (it)

Simetikonas tai meteorizmo simptomams mažinti skirtas medikamentas, vartojamas tik peroraliai, tad vaisto sisteminis poveikis išlieka nežinomas. Pristatome klinikinį atvejį, kai 4 metų pacientei netyčia į veną buvo suleistas simetikonas, sukėlęs kvėpavimo funkcijos sutrikimus, cianozę ir progresuojantį sąmonės sutrikimą. Skubiai pradėjus gydymą būklė įgavo teigiamą dinamiką. Šis atvejis atspindi ypatingai retą įvykį, kadangi vienintelis toks incidentas medicinos literatūroje.

## Introduction

Since its inception in 1940, simethicone has persisted as a prevalent antiflatulent agent in clinical practice. Acting as a surfactant, it facilitates the passage of accumulated gas bubbles. The drug is not systemically absorbed and is thus eliminated via fecal excretion [1–3]. The mechanisms of action and excretion route elucidate the exclusive availability of simethicone in oral formulations, leaving its systemic effects unknown.

## Case Presentation

A 4-year-old female presented with fever and diarrhea, prompting hospital admission. Suspected viral infection led to symptomatic management, including intravenous fluid rehydration and oral simethicone for bloating. The nursing staff facilitated administration of the liquid medication by providing it in a syringe, thereby enhancing convenience for the patient’s mother to administer the medication orally. As bloating worsened, the mother injected simethicone through a peripheral vein catheter. Subsequently, the child exhibited signs of distress, manifested by anxiety, respiratory difficulty, pallor, central and peripheral cyanosis, and rapid deterioration in mental status. Recorded vital signs included a respiratory rate of 22 breaths per minute, and a progressively rising heart rate from 138 to 170 beats per minute. Abdominal distention with absent bowel sounds was noted. Prompt intervention ensued, involving the administration of 9 litres of oxygen via a mask, 20 ml/kg of 0.9% NaCl, and 4 mg of intravenous dexamethasone. The patient was transferred to the intensive care unit (ICU). Arterial blood gas analysis revealed a pH shift from 7.31 to 7.28, indicative of compensated metabolic acidosis, with a serum lactate concentration of 1.2 mmol/l. Coagulation parameters demonstrated no significant elevation, except for fibrinogen at 4.7 g/l (normal range being 2–4 g/l). Other laboratory parameters remained within normal ranges: haemoglobin (>110 mg/l), leukocyte count (5–15,5 *109/l), C-reactive protein (<5 mg/l), and liver enzymes (AST 5-36 U/I, ALT 5-35 U/I). The mother disclosed the inadvertent intravenous administration of simethicone, leading to immediate contact with the Poison Information Centre for guidance. The recommended course of action entailed close monitoring and symptomatic management, comprising administration of 20 mg/kg of paracetamol, 0.2 mg/kg of midazolam, glucose infusions with 10% NaCl at 4 ml/kg/h, and a heparin infusion at 20 U/kg. Following a 40-minute interval, the patient exhibited improved mental status, characterized by increased calmness. There was also a restoration of skin warmth, and subsequent ECG readings indicated no changes. The patient was discharged from the ICU the next day in a fully recovered state.

## Discussion

This case report delineates a rare clinical incident involving intravenous simethicone administration, a phenomenon scarcely encountered by Poison Information Centres. The sole documented case in medical literature dates back to 2003 when Rolf C. Mahne, Johan Damen, and Frank G. A. Jansman reported an incident involving a 62-year-old septic-shock patient in the intensive care unit (ICU) [4]. In both cases, the initial manifestation of adverse effects was respiratory distress. The dyspnea in the 4-year-old patient rapidly progressed, accompanied by visible cyanosis, while the 62-year-old patient experienced a sudden decline in oxygen saturation from 98% to 88%, with corresponding increases in FiO2 from 40% to 100% and PEEP from 10 to 14 cmH20. Additionally, both cases demonstrated elevated serum lactate levels (1.2 mmol/l in the 4-year-old, peaking at 2.9 mmol/l in the 62-year-old 10 hours post-incident.

Crucially, it is imperative to underscore that in the latter case, besides simethicone, a 10 ml oral solution for selective decontamination of the digestive tract was administered. This solution comprised 500 mg of amphotericin B, 100 mg of colistin sulfate, and 80 mg of tobramycin sulfate per 10 ml, in addition to viscosity enhancers carbomer and propylene glycol. While parallels exist between the two cases, discrepancies in clinical presentation, notably the absence of a febrile response in the current case, warrant acknowledgment. Drawing direct correlations poses challenges, particularly given the septic shock status of the 62-year-old patient in the prior case.

The authors of the 2003 report speculated that hydrophobic simethicone might induce pulmonary microvascular occlusion, simulating acute respiratory distress syndrome clinically. Noteworthy findings in the previous case included radiographic evidence of a slight worsening left pleural effusion, and subsequent regression.

## Conclusion

The accidental intravenous administration of simethicone precipitates transient oxygenation impairment. Given the absence of established protocols, this case report contributes valuable insights into the potential effects of intravenous simethicone. The rarity of documented cases emphasizes the imperative for heightened awareness and vigilance among clinicians. Disseminating this knowledge is paramount for informing future clinical practice and interventions.
